# Clinical Effectiveness of Barrier Preparations in the Management of Diaper Dermatitis: A Systematic Review and Meta-Analysis

**DOI:** 10.7759/cureus.102379

**Published:** 2026-01-27

**Authors:** Zainab Z Alkhamis, Muskaan Bhagchandani, Mumin Idris, Yusuf Bhyat, Sara M Alsani, Arfa I Pasha, Ahmed Hesham Atif Ali, Dhuha Ali Eledresi, Alaa Ahmed Mahdi, Fatimah Ridha Almohamedhusain

**Affiliations:** 1 Pediatrics and Child Health, Gulf Medical University, Ajman, ARE; 2 Internal Medicine, Thumbay University Hospital, Ajman, ARE; 3 General Practice, Gulf Medical University, Ajman, ARE; 4 Family Medicine, Dubai Medical College for Girls, Dubai, ARE; 5 Surgery, Sheikh Khalifa Medical City, Abu Dhabi, ARE; 6 Internal Medicine, Ras Al Khaimah Medical and Health Sciences University, Ras Al Khaimah, ARE; 7 General Practice, University of Sharjah, Sharjah, ARE; 8 Medicine, Health and Medical Services (HMS) Al Garhoud Private Hospital, Dubai, ARE; 9 Internal Medicine, Medical University of Warsaw, Warsaw, POL

**Keywords:** barrier creams, barrier preparations, children, clinical effectiveness, diaper dermatitis, diaper rash, emollients, meta-analysis, systematic review, treatment efficacy

## Abstract

The inflammatory skin condition known as diaper dermatitis (DD) often affects infants and preschoolers. Barrier preparations are commonly used in their management, but their effectiveness remains unclear. This systematic review aimed to evaluate the extent to which barrier preparations reduce DD compared to a placebo or other therapies. To gather relevant information, we conducted electronic searches on databases such as Medical Literature Analysis and Retrieval System Online (MEDLINE) via PubMed, Scopus, Web of Science, Cochrane Central Register of Controlled Trials (CENTRAL), and Google Scholar for data extraction. We included studies based on randomized controlled trials (RCTs), quasi-randomized controlled studies, and cohort studies. The collected data were analyzed using RevMan version 5.3 (The Cochrane Collaboration, London, United Kingdom) for Windows. This study included a total of six studies, comprising four randomized controlled trials and two cohort studies. The analysis focused on the incidence rates of dermatitis among both experimental and placebo groups across approximately four studies. A significant difference in dermatitis prevalence was observed between the intervention and placebo groups (odds ratio {ORs} = 0.77; confidence interval {CI} = 0.37-1.61; p = 0.0002), with notable heterogeneity noted (degrees of freedom {df} = 3; I^2^ = 79%). Among the six included studies, clotrimazole emerged as an effective management strategy, demonstrating minimal incidence rates of dermatitis compared to placebo. Additionally, zinc oxide (ZnO) paste showed effectiveness in managing diaper dermatitis among neonates. The use of diaper cream or moisturizers, along with soap, was found to be effective in preventing nappy rash. However, only one study reported no efficacy of petroleum jelly or Vaseline in reducing the severity and incidence of dermatitis symptoms. The effectiveness of barrier preparations in treating DD varies depending on the preparation and study design. Clotrimazole shows potential, especially for suspected fungal infections. To gain a deeper understanding of barrier preparations, identify the factors influencing their efficacy, and explore their role in high-risk patients, additional research is necessary.

## Introduction and background

Diaper dermatitis (DD) is a broad term used to describe the acute inflammatory skin response caused by irritation from urine, feces, moisture, or friction in the diaper area of infants and children. It is also known as diaper rash, nappy rash, or irritant nappy dermatitis and typically occurs at least once during diaper usage [1-4]. This condition affects between 7% and 35% of infants, although some studies report rates as high as 50% at some point, leading to significant discomfort, distress, and disrupted sleep, which can be distressing for both the infant and the caregiver [5]. The prevalence of DD varies significantly across different countries: it ranges from 10% to 20% in the USA [6], 36% in Thailand [7], 14.9% in Germany [8], 1.3%-43.8% in China [9], 67% in Turkey [10], 38.9% in Nigeria [11], and 25% in Japan [12].

The rash seen in DD has multiple causes, stemming from the skin’s response to various systemic and local factors. When diapers are used, the skin’s alkalinity and moisture levels increase significantly. Prolonged exposure to moisture can lead to skin maceration, weakening the stratum corneum’s physical integrity and making it more susceptible to friction from the diaper’s surface. This prolonged moisture also increases the likelihood of developing additional skin damage and other issues due to exposure to irritants, especially urine and fecal lipases and proteases, which are known to be significant contributors to the development of DD [13-15].

Furthermore, frequent skin cleansing accelerates the loss of surface lipids and epidermal cells, disrupting the skin’s acid mantle and compromising its barrier function. Children who experience diarrhea, undergo ostomy takedown, or undergo colon surgery typically exhibit faster gastrointestinal transit, leading to the increased activity of fecal proteases and lipases. Consequently, these children are at a considerably higher risk of developing severe DD [13,14,16].

To address these challenges, the primary focus of management strategies for DD is to minimize inflammation and promote healing, a reassurance for caregivers and healthcare professionals [14,16]. Barrier preparations, such as topical ointments, creams, or pastes, aim to create a physical barrier on the skin’s surface. They have become a cornerstone in treating and preventing this common condition. In the context of DD, these products are designed to protect the skin from irritants such as urine, feces, and diaper friction. Common ingredients in barrier treatments include petrolatum, dimethicone, and zinc oxide (ZnO), each offering unique qualities that reduce friction, decrease irritation, and maintain skin hydration [17,18].

The introduction of disposable diapers, such as Pampers, in 1963 revolutionized diapering practices. Although disposable diapers are widely used (estimated at 80%-90% in affluent nations), they have not eliminated diaper rash [1,19]. Advancements, such as topical barrier creams and superabsorbent fabrics, aim to reduce the frequency and severity of diaper rash. Despite their widespread use, the current understanding of the therapeutic effectiveness of barrier preparations remains limited. Variations in methodology, techniques for applying barrier preparations, and outcome measures make it challenging to draw definitive conclusions about their overall efficacy.

Barrier product selection is often driven more by marketing claims than actual efficacy [20]. This highlights the importance for medical professionals, particularly those working with children, to stay up-to-date on the latest findings on over-the-counter (OTC) barrier therapies for DD. Through a comprehensive and quantitatively synthesized analysis of recent research on barrier preparations in DD, this systematic review and meta-analysis aims to address these limitations. They also strive to bridge knowledge gaps and provide caregivers and healthcare professionals with valuable evidence-based information. The results offer clarity on the role of barrier preparations in treating DD, which can inform therapeutic approaches. Ultimately, a deeper understanding of their efficacy can lead to improved therapy outcomes and better health for infants with this common condition.

## Review

Materials and methods

The recent systematic review adhered to the guidelines outlined in the Preferred Reporting Items for Systematic Reviews and Meta-Analyses (PRISMA) to ensure comprehensive reporting and methodological adherence [21].

Search Strategy

The meta-analysis and systematic review focused on the clinical effectiveness of barrier preparations in managing diaper rash, also known as diaper dermatitis. Relevant research papers were identified using electronic databases, including Medical Literature Analysis and Retrieval System Online (MEDLINE) via PubMed, Scopus, Web of Science, Cochrane Central Register of Controlled Trials (CENTRAL), and Google Scholar, for data extraction. To ensure data authenticity, MeSH keywords such as “Diaper Dermatitis,” “Diaper Rash,” “Nappy Rash,” “Infant Skin Diseases,” “Irritant Dermatitis,” “Zinc Oxide,” and “Petroleum Jelly or Ointment” were used. The research timeline spanned from January 2004 to January 2024, gathering data from the past 20 years.

Eligibility Criteria

Inclusion criteria: Following the search across the mentioned electronic databases, our research team utilized predefined eligibility criteria to screen research articles for inclusion in the recent meta-analysis and systematic review. Specifically, we selected articles that met the following criteria: 1) addressed management strategies or barrier preparations; 2) involved a study population comprising infants and toddlers aged 0-2 years; 3) discussed nappy rash, diaper dermatitis, or diaper rash; 4) were based on randomized controlled trials (RCTs), quasi-randomized control studies, or cohort studies; and 5) were published in English with full-text availability [22].

Exclusion criteria: Excluded studies exhibited the following characteristics: 1) focused on management strategies for different types of rashes; 2) addressed populations outside the age range of 0-2 years old; 3) encompassed populations without diaper dermatitis or nappy rash; 4) were systematic reviews, meta-analyses, literature reviews, observational studies, scoping reviews, conference papers, or letters; and 5) were published in languages other than English (such as Chinese, Arabic, Spanish, and German) or were duplicate publications/non-full-text papers.

Data Extraction and Efficacy Measures

Data entry and processing were conducted using a standardized Excel sheet (Microsoft Corp., Redmond, WA), with two reviewers responsible for extracting data from the included studies. The extracted data encompassed several domains, including study years, the country of origin, study follow-up duration, sample sizes, types of barrier preparations used for management, and primary outcomes from the selected articles. The reviewers independently extracted data from the articles included, resolving any discrepancies through discussion.

Types of Intervention and Primary Outcomes

The recent systematic review and meta-analysis identified significant barrier types for managing diaper dermatitis among infants: soap and water, zinc oxide paste, petroleum jelly (Vaseline), lanolin, and hydrozole ointment [22]. The primary outcomes of the meta-analysis included preventing nappy dermatitis, the number of infants showing a decrease in nappy dermatitis severity, and the reduction in nappy dermatitis duration (measured in days). The severity of the rash was assessed using the following scale: severe (skin breakdown), mild/moderate (any rash present), and absent (no rash).

Risk of Bias Assessment

To assess the risk of bias in the included RCTs, we employed the Cochrane risk of bias assessment tool, which evaluates bias across seven domains: (a) allocation concealment, (b) selection bias or random sequence generation, (c) performance bias or blinding of participants and personnel, (d) detection bias or blinding of outcome assessment, (e) selective bias or selective reporting, and other biases [23]. Each domain’s score was categorized as low risk, high risk, or unclear.

For other cohort studies, we used the Joanna Briggs Institute (JBI) critical appraisal checklist to assess the methodological quality of the included prospective cohort studies. Additionally, we evaluated the methodological quality of included cross-sectional studies and the strategies they employed to address and minimize bias using the JBI critical assessment instrument [24].

Statistical Analysis

The statistical analysis of data from studies included in the recent meta-analysis and systematic review was conducted using RevMan 5.3 software (The Cochrane Collaboration, London, United Kingdom) [21]. A p-value of <0.05 was considered statistically significant when presenting results as odds ratios (ORs) with 95% confidence intervals (CIs). Additionally, heterogeneity was assessed using the Q test and I^2^ statistics. A fixed effects model was used when no significant heterogeneity was detected, while a random effects model was applied otherwise.

Results

Included Studies

**Figure 1:**
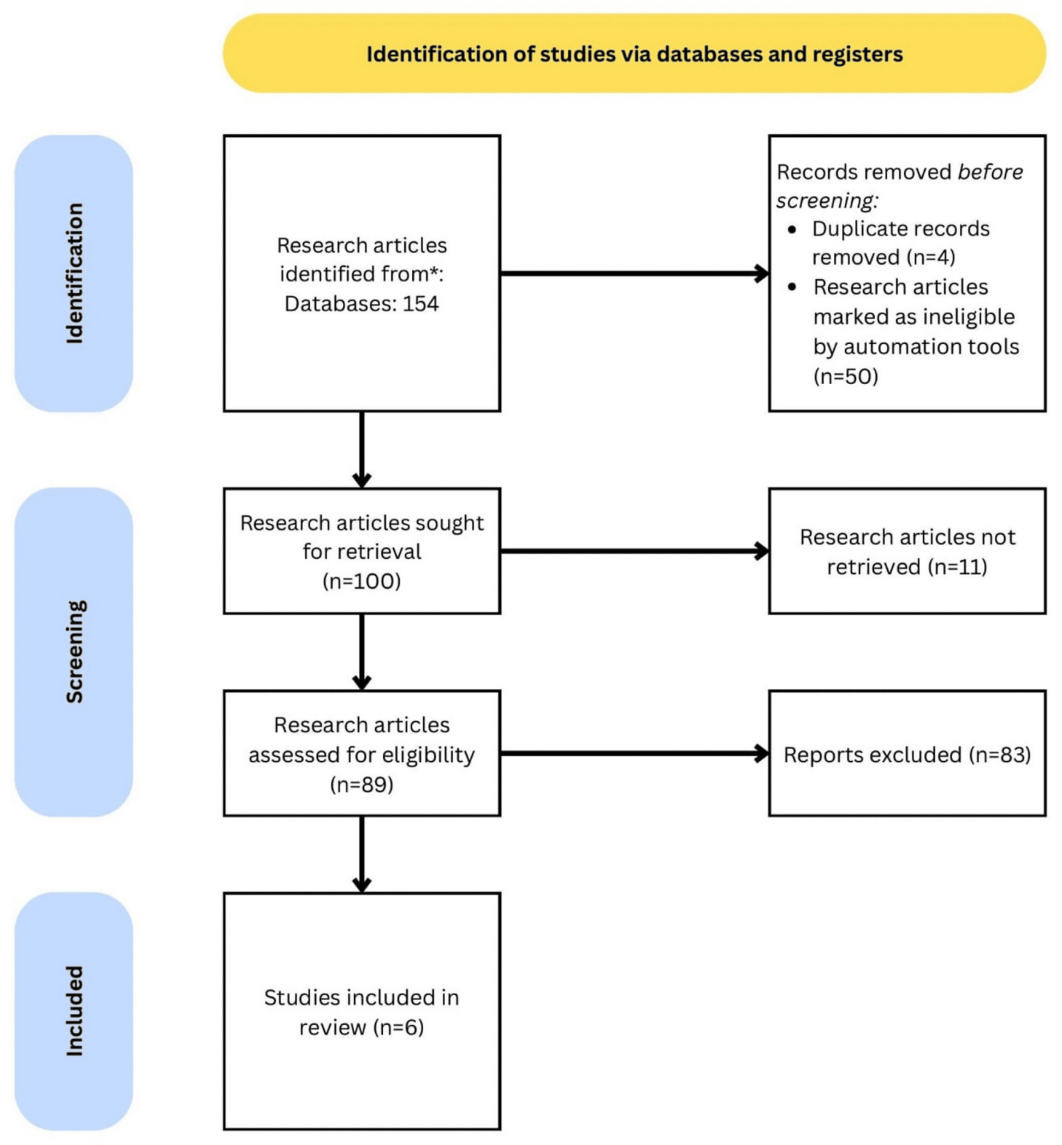
Screening and selection of the included studies by PRISMA guidelines *Research articles were retrieved from the following databases: MEDLINE via PubMed, Scopus, Web of Science, Cochrane Central Register of Controlled Trials (CENTRAL), and Google Scholar, including randomized controlled trials, quasi-randomized controlled studies, and cohort studies PRISMA, Preferred Reporting Items for Systematic Reviews and Meta-Analyses; MEDLINE, Medical Literature Analysis and Retrieval System Online

The selection and screening of research papers on the clinical effectiveness of barrier preparations for managing diaper dermatitis were conducted according to the PRISMA guidelines in the recent meta-analysis. Initially, 154 research articles were identified from three electronic databases using the specified search strategy. Fifty-four articles were removed due to duplicate records or ineligibility, and 11 articles could not be retrieved. Following the PRISMA guidelines, 89 papers were then screened, and six research articles were included after applying the exclusion criteria (Figure 1) [24].

Risk of Bias Assessment

**Figure 2:**
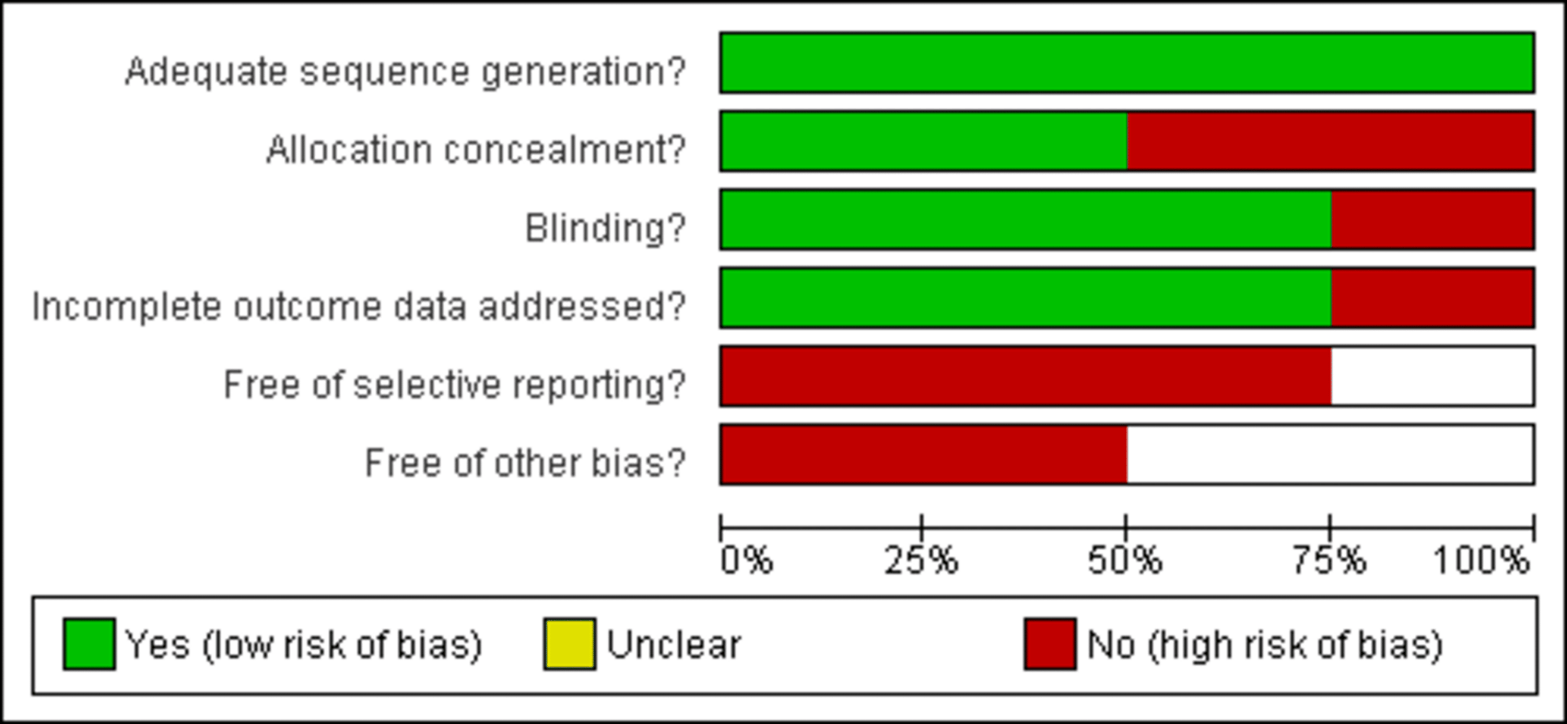
Risk bias of the included studies

**Figure 3:**
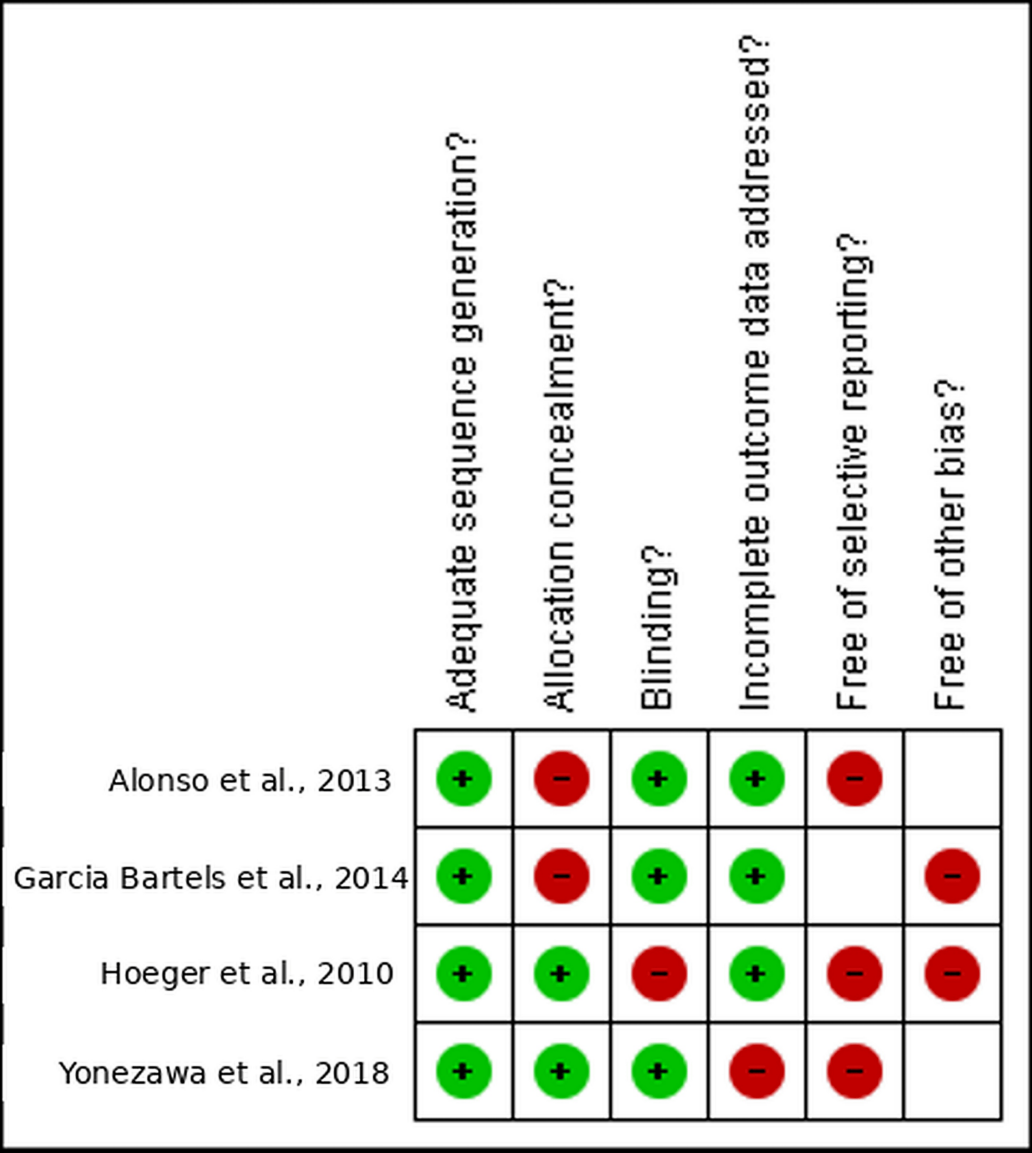
Visual representation of risk bias summary for the included studies Data adapted from Alonso et al. [25], Yonezawa et al. [26], Garcia Bartels et al. [27], and Hoeger et al. [28]

Out of the six studies included, four were randomized controlled trials evaluated using the Cochrane Library tool. Among these, three out of four were deemed to have low to moderate risk [25-27], while one study was classified as high risk [28], as illustrated in Figure 2 and Figure 3. The remaining two studies were cohort studies, and their methodological quality assessment was conducted using the JBI criteria (Table 1) [29,30].

**Table 1:** Checklist of JBI for quality assessment JBI: Joanna Briggs Institute

Questions	Bonifaz et al., 2013 [30]	Chaithirayanon et al., 2016 [29]
Were the two groups similar and recruited from the same population?	Yes	No
Were the exposures measured similarly to assign people to both the exposed and unexposed groups?	Yes	Yes
Was the exposure measured in a valid and reliable way?	Yes	No
Were confounding factors identified?	No	Yes
Were strategies to deal with confounding factors stated?	Yes	No
Was the follow-up time reported and sufficient to be long enough for outcomes to occur?	No	Yes
Was an appropriate statistical analysis used?	No	Yes
Were strategies to address incomplete follow-up utilized?	Yes	No
Was a follow-up completed, and if not, were the reasons for failure to follow-up described and explored?	Yes	Unclear
Were the outcomes measured in a valid and reliable way?	Yes	Unclear
Were the groups/participants free of the outcome at the start of the study?	No	Yes
Were strategies to address incomplete follow-up utilized?	No	Yes

Characteristics of the Included Studies

The articles included in the recent systematic review and meta-analysis were published between 2004 and 2024. All included trials were randomized controlled trials. To produce heterogeneity of results, the trials belong to five different countries: two in Germany [26,27], one in Spain [25], one in Japan [26], one in Thailand [29], and one in Mexico [30]. Table 2 provides detailed characteristics of the included studies.

**Table 2:** Characteristics of the included studies

Author and year	Country	Study population and sample size	Age of the children	Type of design	Type of barrier preparation	Number of infants with a decrease in the severity of nappy dermatitis	Prevention of diaper dermatitis
Alonso et al., 2013 [25]	Spain	426 neonate population: 213 in experimental neonates and 213 in control	18 months	Randomized controlled trial	Petroleum jelly	Petroleum jelly (Vaseline group): 177; control group: 166	Not significantly lowered
Yonezawa et al., 2018 [26]	Japan	227 babies: 113 in the experimental group and 114 in the control	One week and three months	Randomized controlled trial	Water and soap	Water and soap: 67; control: 48	Improved skin dermatitis effectively
Hoeger et al., 2010 [28]	Germany	96 infants: 46 in the zinc oxide paste group and 45 in clotrimazole	6-18 months	Randomized controlled trial	Zinc oxide paste and clotrimazole (hydrozole)	Zinc oxide paste: 21; clotrimazole: 30	Both improved symptoms, but the efficacy of zinc oxide paste is notably low than clotrimazole (hydrozole)
Chaithirayanon et al., 2016 [29]	Thailand	50 infants: 25 in zinc oxide and 25 in talcum	6-12 months	Cohort study	Talcum and zinc oxide cream	Recovery time: 3.7 + 3.3 days in the zinc oxide group and 2.7 + 0.5 days in talcum and 23 in the zinc oxide group and 21 in talcum	Zinc oxide was effective in treating symptoms with less adverse events
Bonifaz et al., 2013 [30]	Mexico	27 infants	2-22 months	Prospective study	Clotrimazole	24 recovered out of 27	Clotrimazole is effective for disease management
Garcia Bartels et al., 2014 [27]	Germany	89 healthy infants: 58 with cream of clotrimazole and 31 with wet wipes	Nine months	Randomized controlled trial	Water and moisturized wipes	55 recovered; 24 recovered	Clotrimazole is more effective than wet wipes or clothes

Infants With Reduced Symptoms

**Figure 4:**
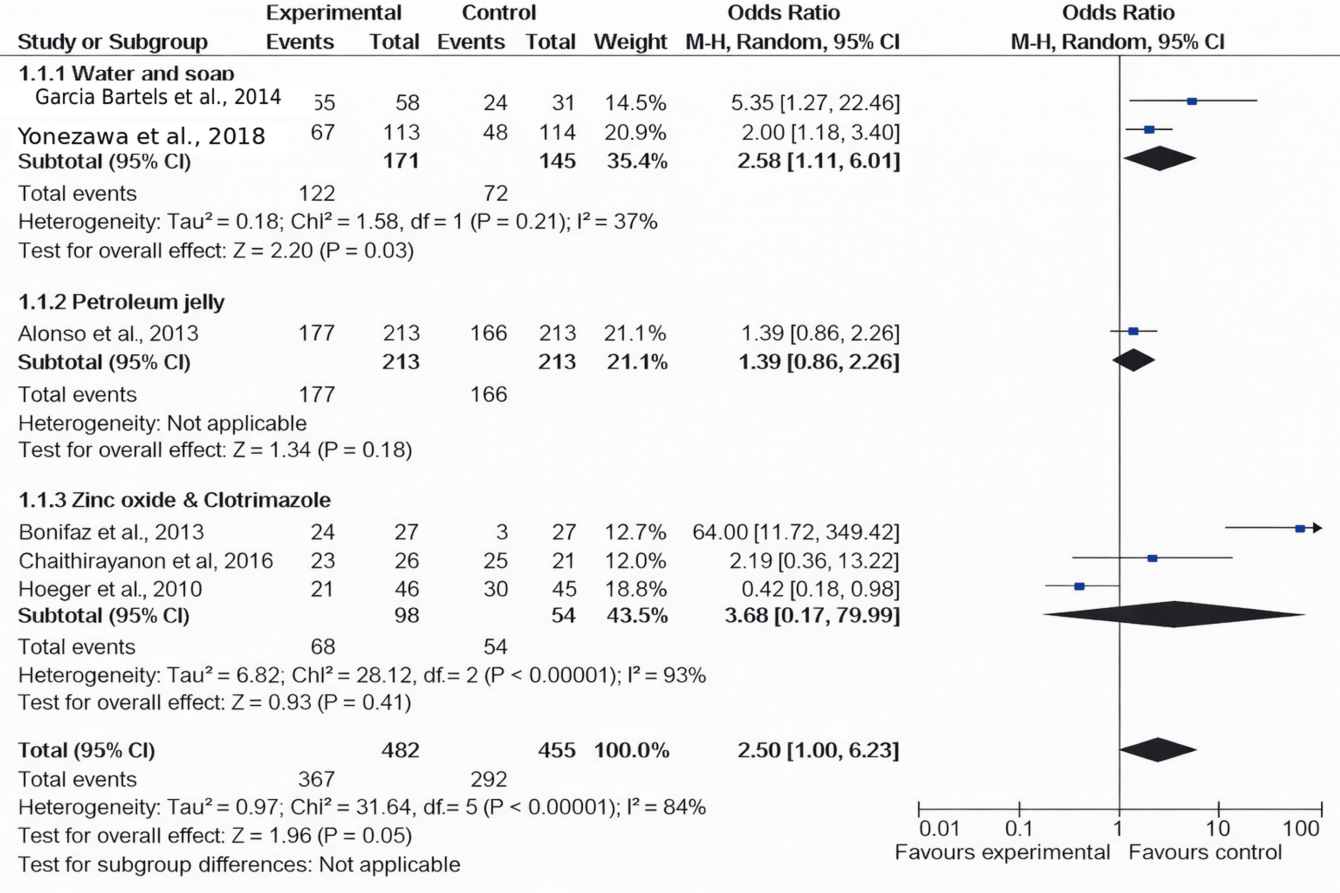
Forest plot of the incidence of dermatitis among the experimental and placebo groups Data adapted from Alonso et al. [25], Yonezawa et al. [26], Garcia Bartels et al. [27], Hoeger et al. [28], Chaithirayanon et al. [29], and Bonifaz et al. [30] CI, confidence interval; df, degrees of freedom

**Figure 5:**
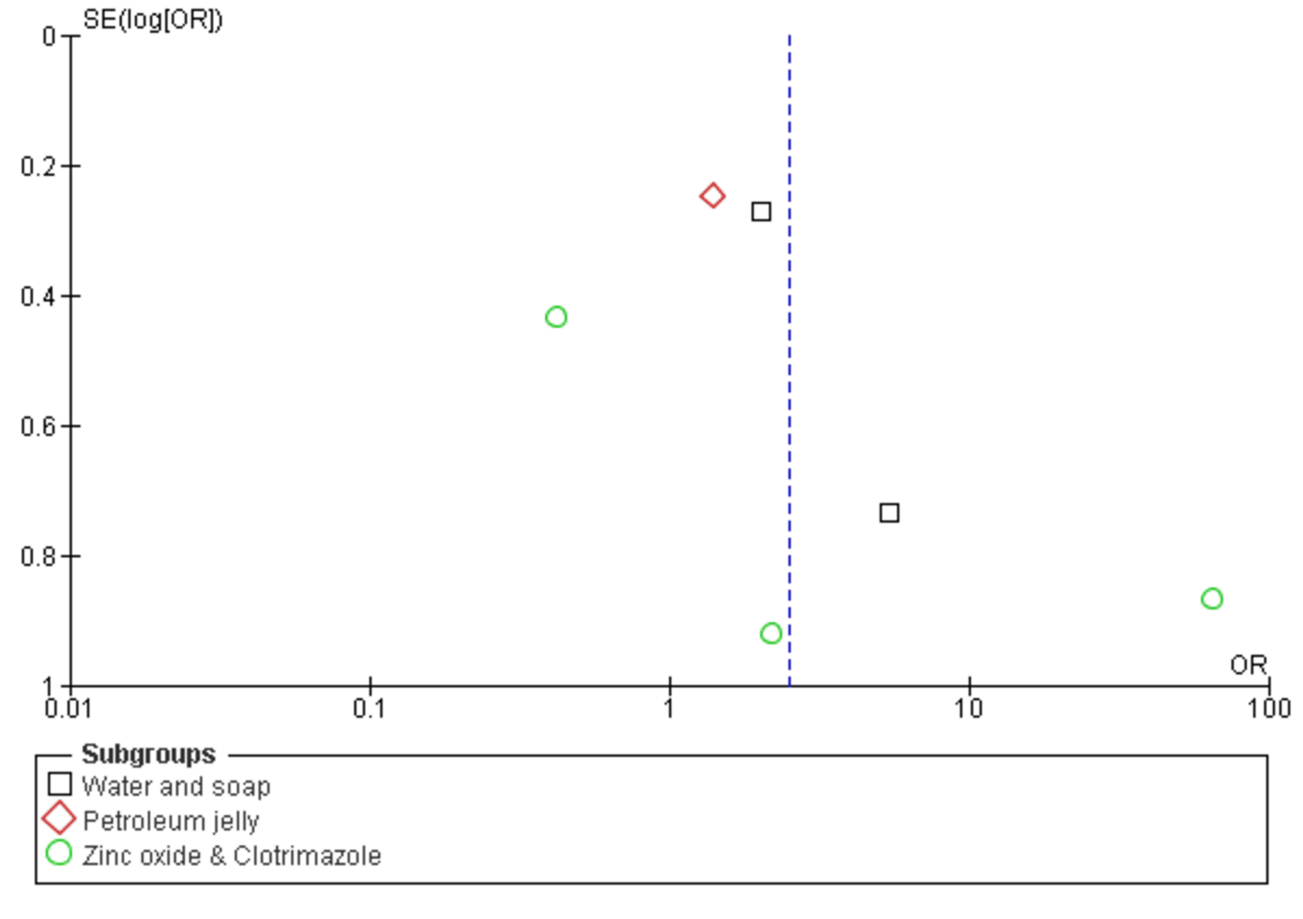
The funnel plot of the incidence of dermatitis among the experimental and placebo groups SE, standard error; OR, odds ratio

Among the six included studies, approximately four examined the decrease in dermatitis incidence rates in both the experimental and placebo groups [25-28]. A significant difference in the prevalence of dermatitis was noted between the intervention and placebo groups (odds ratio = 0.77; CI = 0.37-1.61; p = 0.0002), and heterogeneity was observed (degrees of freedom {df} = 3; I^2^ = 79%), as illustrated in Figure 4 and Figure 5.

Prevention of Diaper Dermatitis

Among the six included studies, clotrimazole emerged as an effective management strategy, with a lower incidence of dermatitis compared to placebo [27,28,30]. Additionally, zinc oxide paste demonstrated effectiveness in managing diaper dermatitis among neonates or infants. Regarding prevention strategies, the use of diaper cream or moisturizers, along with soap, proved effective in controlling the incidence of nappy rash [28,29]. However, only one study reported no efficacy of petroleum jelly or Vaseline in reducing the severity and incidence of dermatitis symptoms [25].

Discussion

Diaper dermatitis (DD) remains a widespread concern for children and caregivers globally. Since its initial documented description in 1877, numerous studies have been undertaken to elucidate the condition’s causes, the most efficacious treatment approaches, and methods to prevent exacerbation. This systematic study aimed to comprehensively assess over-the-counter (OTC) barrier treatments for DD. By meticulously reviewing the literature, we aimed to furnish valuable insights to healthcare providers and families. DD’s peak incidence typically falls between nine and 12 months, making it one of early childhood’s most prevalent dermatological disorders, with an incidence ranging from 7% to 35% [1,19]. Consequently, it remains a concern shared by both caregivers and healthcare professionals. This concern has spurred a substantial industry, as evidenced by the array of products available for DD treatment and/or prevention, the multitude of online platforms dedicated to the topic, and the frequency of medical consultations [31].

Numerous products claim to protect the skin from damage caused by exposure to urine and feces. Compared to placebo, the meta-analysis showed a moderate reduction in DD incidence. However, significant variability was observed, suggesting that efficacy may vary depending on the barrier preparation and study methodology. The investigations explored a range of barrier preparations, including clotrimazole, petroleum jelly, water and soap, zinc oxide paste, and nappy creams/moisturizers [25-30].

Experts recommend barrier creams containing petrolatum or zinc oxide for their ability to protect the skin from moisture and potentially reduce the severity of DD [32]. In our studies, Hoeger et al. [28] found that zinc oxide (ZnO) paste was less effective than clotrimazole, whereas another study reported quicker recovery times than with talcum powder [29]. However, methodological quality assessment revealed missing critical information, raising the potential for bias in the findings. It is unclear whether this resulted from inadequate reporting rather than poor design, so conclusions are supported by low-level evidence (grade C), which urges caution in implementation [28]. In a separate study, the 2% eosin group showed significantly higher rates of full and partial healing compared to a corticosteroid and ZnO paste. ZnO paste also promoted healing, with 22% achieving complete healing and 44% partial healing [33].

The potential preventive effects of water and soap on DD have not been definitively demonstrated in published research, although a Japanese study concluded that their practical use improves DD [26]. There is insufficient evidence to suggest that these practices have a positive impact on the physiological skin parameters necessary for maintaining healthy skin in the diaper area. Antifungal medications such as nystatin and clotrimazole are recommended when candidal infections exacerbate the irritation of dermatitis [34]. Clotrimazole, in particular, emerged as an effective treatment for DD across multiple studies, with minimal incidence rates compared to placebo. This suggests that clotrimazole may be a beneficial treatment option, especially when fungal colonization is suspected [27,28,30]. Hoeger et al. compared the safety and effectiveness of two distinct antifungal pastes in infants with DD, finding that clotrimazole was more effective [28]. Bonifaz et al. indicated that DD candidiasis might be effectively treated with 2% tetraconazole cream, indirectly supporting the utility of antifungal medications in DD management [30]. Garcia Bartels et al. investigated how wet wipes and diaper cream affected an infant’s skin barrier, revealing that clotrimazole was superior to clothing or wet wipes for managing DD [27]. These studies, with lower dermatitis incidence rates than placebo, suggest that clotrimazole is an effective therapeutic strategy for DD.

Nearly 20 years have passed since Gupta and Bluhm investigated the regular use of petroleum-based barrier cream at each diaper change to prevent DD in high-risk children [35]. Their findings suggested that if the skin in the diaper area remained intact during cream application, using a petroleum-based cream was beneficial for preventing DD. However, more extensive controlled studies are needed due to the study’s limited sample size and the persistent issue of DD in this high-risk group. The inability to conduct a meta-analysis, even within the general newborn population, was due to differences in study design, resulting in narrowly focused conclusions. Similarly, a study from Spain found no significant advantage of petroleum jelly (Vaseline) in reducing the frequency or intensity of DD symptoms, challenging the widespread belief in its prophylactic role [25]. Further investigation is needed to determine the exact role of petroleum jelly in the treatment of DD.

Despite these findings, several limitations exist. The review may have overlooked crucial information from alternative study designs, such as cohort studies, as it primarily focused on RCTs, which are considered the gold standard of evidence. Moreover, the observed heterogeneity highlights the need for further investigation into the variables that influence the efficacy of barrier preparations. While the review identified potential benefits of ZnO and clotrimazole, the effectiveness of other barrier preparations, such as natural or organic alternatives, remains unknown. More research is necessary to determine the optimal application frequency and duration for various barrier preparations. The severity of DD may also impact the effectiveness of barrier preparations, and individual differences in skin type and potential allergies to the components of barrier treatments must be taken into account. Exploring how barrier preparations interact with regular diaper changes, hygiene practices, or other topical treatments may provide valuable insights.

## Conclusions

This systematic review concluded that barrier preparations are effective in managing diaper dermatitis in infants. While the analysis indicated a moderate overall effect compared to placebo, there were notable variations across studies. Clotrimazole was identified as a promising barrier treatment for diaper dermatitis, particularly in suspected fungal infections. Although zinc oxide paste showed potential for treating diaper dermatitis, further research is needed to determine its optimal application. Petroleum jelly appeared to provide no significant benefits. Future investigations should include cost-effectiveness assessments and consider integrating barrier methods with other management strategies.
